# Far-lateral Disc Herniation Treated by Lateral Lumbar Interbody Fusion without Complete Fragment Excision: A Case Report and Review of the Literature

**DOI:** 10.7759/cureus.3404

**Published:** 2018-10-02

**Authors:** Colin M Haines, Rahul G Samtani, James T Bernatz, Mustafa Abugideiri, Joseph R O'Brien

**Affiliations:** 1 Orthopaedics, Virginia Spine Institute, Reston, USA; 2 Orthopaedics, University of Wisconsin Hospital and Clinics, Madison, USA; 3 Orthopaedics, University of Wisconsin School of Medicine and Public Health, Madison, USA; 4 Radiation Oncology, Emory University, Atlanta, USA; 5 Orthopaedics, Washington Spine and Scoliosis Institute, Bethesda, USA

**Keywords:** xlif, llif, far-lateral herniation, lumbar fusion

## Abstract

Symptomatic far-lateral lumbar disc herniation is a less common causes of lumbar radiculopathy than paracentral or central disc herniation. Treatment of far-lateral disc herniation with a retroperitoneal, transpsoas approach and disc fragment excision has been described. However, treatment of far-lateral disc herniation using lateral lumbar interbody fusion (LLIF) without neural manipulation has not been described. We report one case in which symptom resolution was accomplished via indirect decompression with anterior column support via LLIF without disc fragment excision and review the current literature. The patient noted immediate relief of his preoperative leg pain in the recovery room and ambulation began the same day. Narcotics were effective in treating his incisional pain and mild back pain. The patient was seen two weeks postoperatively and he had stopped all narcotics. At six weeks, the patient continued to have significant improvement and was able to take hour-long walks. At five months, the patient did not have any pain and continued to have improvement in his left quadriceps strength. Minimally invasive lateral lumbar interbody fusion has allowed surgeons to provide both direct and indirect neural decompression through a retroperitoneal approach. This technique may be ideal for far-lateral disc herniation as it also allows a lateral visualization of the herniation without bony, posterior muscular, or ligamentous disruption.

## Introduction

Lumbar disc herniations are a common cause of lower extremity radiculopathy. However, only 7–12% of disc herniations are in the far-lateral region, or lateral to the neuroforamen [1-5]. These herniations can be difficult to treat from a posterior approach because of the anatomy of the surrounding facet joints. Traditionally, this pathology has been accessed posteriorly via a paramedian, extraforaminal approach [1,6-8]. In a recent publication, Madhok and Kanter described a lateral, retroperitoneal approach without fusion to excise the compressive disc fragment resulting in successful treatment of two cases of far-lateral disc herniation [3]. We present one case in which clinical relief from a far-lateral disc hernation was accomplished using a lateral lumbar interbody fusion (LLIF) approach without direct decompression.

## Case presentation

**Figure 1:**
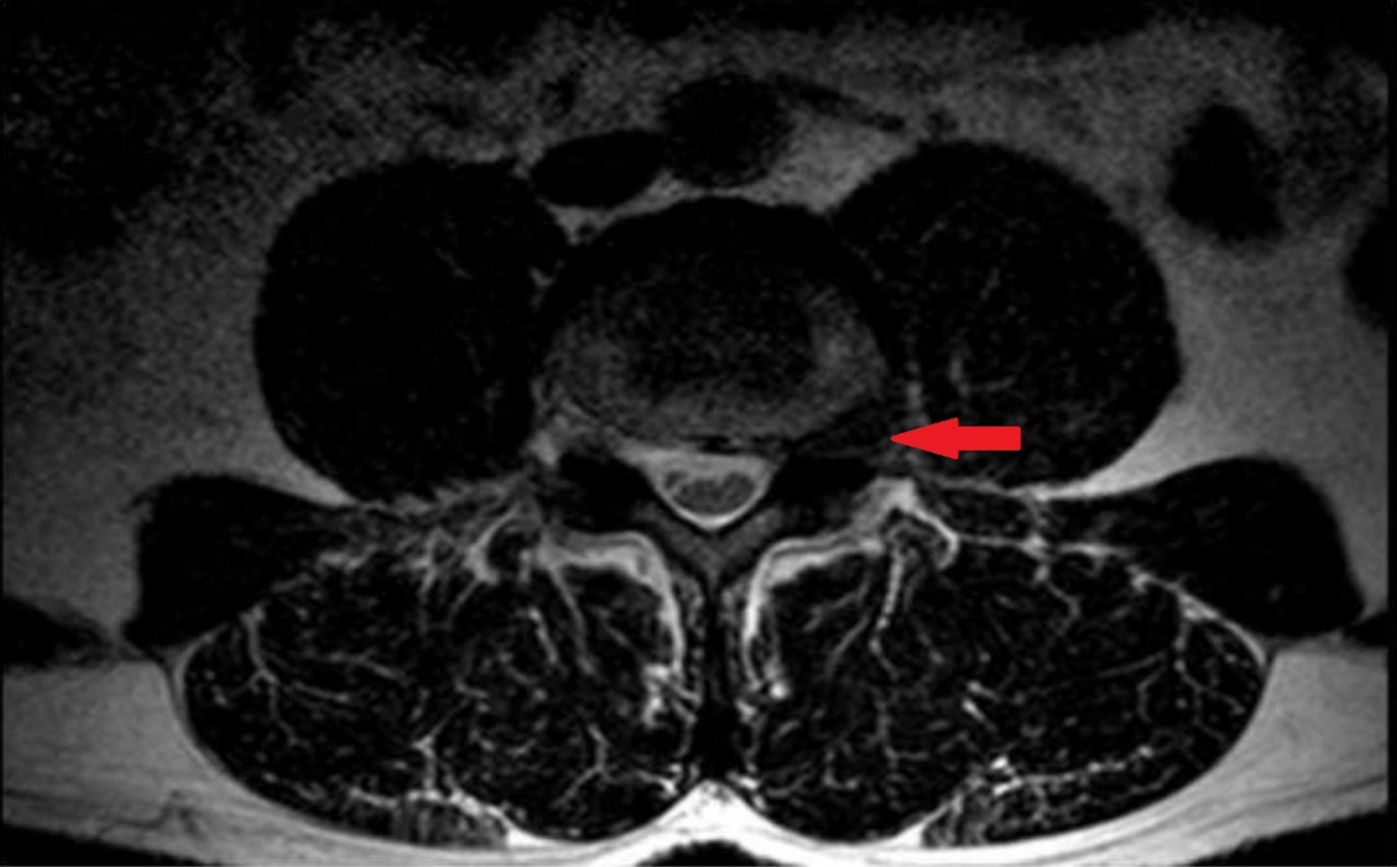
Axial non-contrast T2-weighted magnetic resonance image demonstrating a left far-lateral disc herniation at L3-4 (red arrow).

A 49-year-old male presented to our clinic with left medial thigh pain, quadriceps atrophy, and weakness. Non-surgical management consisted of a trial of oral narcotics, physical therapy, and epidural steroid injections. Magnetic resonance imaging revealed a left sided L3-4 far-lateral disc herniation with compression of the left L3 nerve root (Figure 1).

The patient was brought to the operating room and positioned in a left lateral decubitus position (right side up). Intraoperative fluoroscopy was used to localize a 3.5 cm incision over the L3-L4 disc space. The skin was incised, then blunt dissection was used to divide the external oblique, internal oblique, and transversus abdominus. The retroperitinum was visualized and endoscopic kitners were used to sweep the fat off the surface of the psoas. Subtotal discectomy was performed with preparation of the endplates. A size 10 mm x 18 mm x 55 mm cage was filled with bone graft and a small bone morphogenic protein (Infuse, Medtronic). The wound was irrigated and closed in a standard fashion.

**Figure 2:**
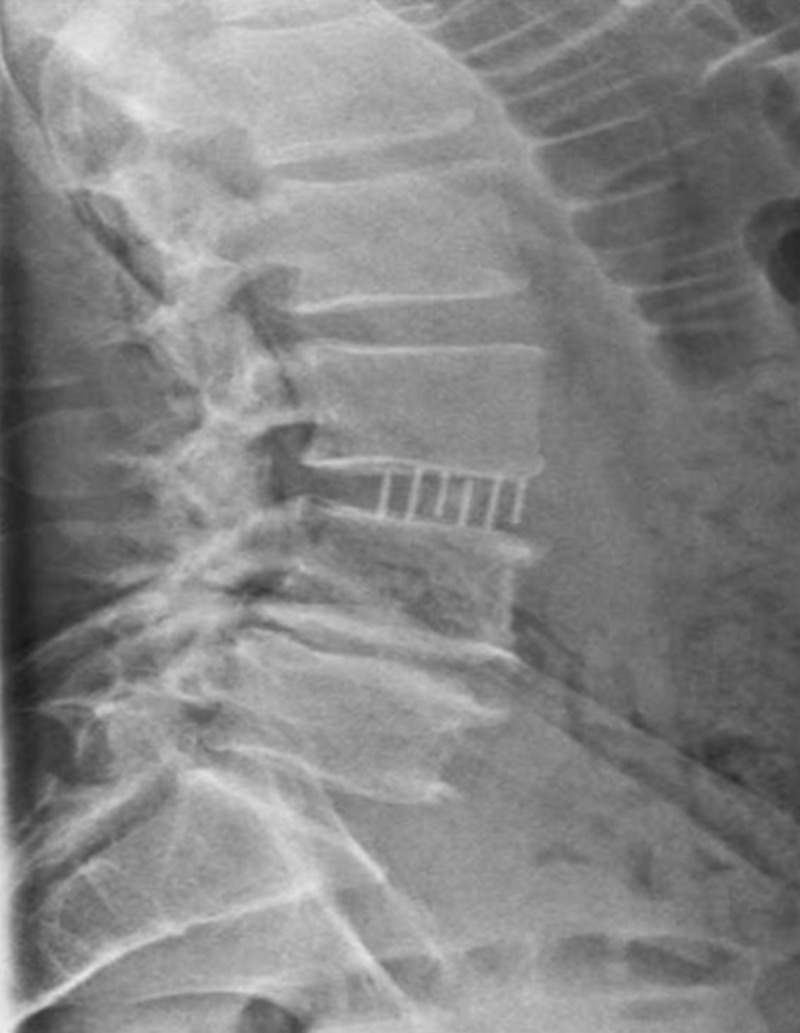
Six-month post-operative lateral standing radiograph demonstrating lateral interbody cage placement.

The patient noted immediate relief of his preoperative leg pain in the recovery room and ambulation began the same day. Narcotic pain relievers (oxycodone) were effective in treating his incisional pain and mild back pain. The patient was seen at two weeks postoperative and he had stopped all narcotic pain relievers. At six weeks, the patient continued to have significant improvement and was able to take hour-long walks. At five months, the patient did not have any pain and continued to have improvement in his left quadriceps strength. Six-month post-operative radiographs demonstrated stable interbody cage positioning without signs of subsidence (Figure 2).

## Discussion

The prevalence, symptomatology, and pathoanatomy of far-lateral disc herniations were first reported by Abdullah et al. [1]. Typically, the exiting nerve root is affected by the disc herniation, in contrast to more midline herniations which affect the traversing nerve root. Discectomy for disc herniation is an accepted and effective treatment for neurologic symptoms resulting from disc herniation [9]. Generally, hemilaminotomy is performed with intra-canal work to perform lumbar discectomy. In far-lateral discectomy, hemilaminotomy may not allow access to the herniation in the extra-foraminal zone.

Reported methods to treat far-lateral herniations include complete facetectomy, the intertransverse technique, the pars technique, and the extraforaminal technique [2-3]. Successful surgical results have been reported using lateral muscle splitting approaches with pars and intertransverse ligament removal to access the affected disc [4,10]. Pitfalls of such approaches included increased postoperative pain from paraspinal muscle dissection as well as the potential for instability if bony resection was performed [3].

More recently, anterolateral approaches have allowed surgeons to visualize intervertebral discs using a minimally invasive approach [11]. The minimally invasive transpsoas approach (LLIF) may be ideal for far-lateral herniations as it also allows a lateral visualization of the herniation without bony or ligamentous disruption. In 2010, a report of the LLIF approach for far-lateral disc fragment excision was published [3]. The authors utilized the LLIF tubular retractor to access the disc using a trans-psoas approach in which fusion was not performed. The patients in the report experienced complete symptom relief postoperatively. In our case, direct decompression was not performed. Instead, LLIF was performed with interbody fusion, and indirect decompression provided excellent and durable pain relief.

In general, the LLIF procedure has been found to be both efficacious and safe, improving radiculopathic symptoms by indirectly increasing neuroforaminal height by 35%, or 36.2 mm squared [12]. In 2010, Youssef et al. retrospectively analyzed 84 patients with this procedure. Eighty-one percent had radiographic fusion at an average follow-up 15.7 months and 92% had stopped all narcotics by six months. The patients had 77% improvement in the visual analog scale as well as a 55% improvement in the Oswestry disability index, both of which were statistically significant [13]. Cage position, after the disc space has been prepared, has also been examined. The posterior disc height increases the most when the cage has been placed in the posterior aspect of the disc as compared to anterior insertion. However, implant placement has not been shown to correlate with clinical parameters. In fact, anterior cage insertion had a trend towards improved symptoms but this did not reach statistical significance [12]. This finding may potentially be due to overly posterior cage placement resulting in neural compression but this has not been verified.

Complications from the anterolateral approach have been reported from 2% up to 60% [14-16], depending on the authors’ definition of an adverse outcome. The most common event is postoperative thigh symptoms which are usually transient, reported from 1% to 60%. This is more commonly from blunt dissection through the psoas or genitofemoral nerve irritation. More serious complications have been reported as well, including a 4.8% femoral nerve injury at the L4-5 level.

Minimally invasive lateral lumbar interbody fusion has allowed surgeons to provide both direct and indirect neural decompression through a retroperitoneal approach. This technique may be ideal for far-lateral herniations as it also allows a lateral visualization of the herniation without bony, posterior muscular, or ligamentous disruption.

## Conclusions

Minimally invasive lateral lumbar interbody fusion has allowed surgeons to provide both direct and indirect neural decompression through a retroperitoneal approach. This technique may be ideal for far-lateral herniations as it also allows a lateral visualization of the herniation without bony, posterior muscular, or ligamentous disruption.
